# Effects of *Carissa carandas* Linn. Fruit, Pulp, Leaf, and Seed on Oxidation, Inflammation, Tyrosinase, Matrix Metalloproteinase, Elastase, and Hyaluronidase Inhibition

**DOI:** 10.3390/antiox10091345

**Published:** 2021-08-25

**Authors:** Waranya Neimkhum, Songyot Anuchapreeda, Wei-Chao Lin, Shang-Chian Lue, Kuan-Han Lee, Wantida Chaiyana

**Affiliations:** 1 Department of Pharmaceutical Science, Faculty of Pharmacy, Chiang Mai University, Chiang Mai 50200, Thailand; waranya_ne@cmu.ac.th; 2 Division of Clinical Microscopy, Department of Medical Technology, Faculty of Associated Medical Sciences, Chiang Mai University, Chiang Mai 50200, Thailand; songyot.anuch@cmu.ac.th; 3 Research Center of Pharmaceutical Nanotechnology, Chiang Mai University, Chiang Mai 50200, Thailand; 4 Department of Cosmetic Science and Institute of Cosmetic Science, Chia Nan University of Pharmacy and Science, Tainan 71710, Taiwan; weilin@mail.cnu.edu.tw (W.-C.L.); myluemy@mail.cnu.edu.tw (S.-C.L.); 5 Department of Pharmacy, Chia Nan University of Pharmacy and Science, Tainan 71710, Taiwan

**Keywords:** *Carissa carandas*, ursolic acid, antioxidant, anti-tyrosinase, matrix metalloproteinases, nuclear factor-kappa B, interleukin, human epidermal keratinocyte line

## Abstract

In this study, the potential of *Carissa carandas* Linn. as a natural anti-aging, antioxidant, and skin whitening agent was studied. Various parts of *C. carandas*, including fruit, leaf, seed, and pulp were sequentially extracted by maceration using *n*-hexane, ethyl acetate, and ethanol, respectively. High-performance liquid chromatography, Folin–Ciocalteu, and Dowd method were used to investigate their chemical compositions. The inhibitory activities of oxidation process, matrix metalloproteinases (MMPs), elastase, hyaluronidase, and tyrosinase were analyzed. Cytotoxicity was determined by 3-(4,5-dimethylthiazol-2-yl)-2,5 diphenyl tetrazolium bromide assay in a human epidermal keratinocyte line (HaCaT). The results exhibited that ethyl acetate could extract the most ursolic acid from *C. carandas*, while ethanol could extract the most phenolics and flavonoids. The leaf extract had the highest content of ursolic acid, phenolics, and flavonoids. The leaf extracted with ethyl acetate (AL) had the highest ursolic acid content (411.8 mg/g extract) and inhibited MMP-1, NF-kappa B, and tyrosinase activity the most. Ursolic acid has been proposed as a key component in these biological activities. Although several *C. carandas* extracts are beneficial to human skin, AL has been proposed for use in cosmetics and cosmeceuticals due to its superior anti-wrinkle, anti-inflammation, and whitening properties.

## 1. Introduction

*Carissa carandas* Linn. is a fruit-bearing plant that grows as a tiny shrub in the Apocynaceae family that is widely distributed in subtropical and tropical regions and has been used for centuries as a medicinal herb in the Ayurvedic, Unani, and Homeopathic systems [1]. In ethnomedicine, different parts of *C. carandas* have been used to treat anorexia, asthma, brain disease, constipation, cough, diarrhea, epilepsy, fever, leprosy, malaria, myopathic spams, pain, pharyngitis, scabies, and seizures [1]. *C. carandas* has been observed to possess a wide spectrum of phytochemical constituents that varied in each part of the plant and result in various biological activities. Tannin, steroidal glycosides, phenolic compounds, and triterpenoidal constituents are abundant in the leaves [2,3]. Several volatile compounds, as well as carissone and carindone, are present in the fruits [1] while the seeds contain fatty acids such as linoleic acid, oleic acid, palmitic acid, and stearic acid [4]. Therefore, all parts of *C. carandas* would be beneficial for human health and would have a potential to be used for anti-skin-aging. Since *C. carandas* contained a wide range of phytochemical constituents, from the lipophilic (e.g., fatty acids) to the hydrophilic (e.g., phenolic compounds), the biological activities of the extracts would vary depending on the polarity of the solvents used in the extraction process.

The methanolic extracts of *C. carandas* leaves have been reported for a potent inhibition potential on DNA damage, oxidation, and inflammatory process [3]. Aside from the leaves, the fruits of *C. carandas* displayed strong anti-inflammatory activity in rats with carrageenan-induced hind paw edema [5]. The 23-hydroxy ursolic acid, a potent anti-inflammatory agent found in *C. carandas*, strongly inhibited the production of reactive oxygen species (ROS) from human whole blood phagocytes [6]. Since ROS is implicated with both intrinsic and extrinsic factors, it is thought to play a crucial role in human skin aging [7]. ROS is a crucial factor in the transcription factors activation that result in the release of pro-inflammatory cytokines and the development of matrix metalloproteinase (MPP), inducing cellular senescence and aging in both the epidermis and dermis layers [8]. Furthermore, ROS directly degraded hyaluronan and activated tyrosinase, resulting in symptoms of aging such as coarseness, sallow discoloration, telangiectasia, wrinkling, and abnormal pigmentation, as well as a host of benign, malignant neoplasms, and premalignant [9].

Prevention of oxidative stress, inflammation, and extracellular matrix (ECM) remodeling were purposed as the strategies for anti-skin-aging [7]. Regarding a variety of phytoconstituents of *C. carandas*, the hypothesis of the study is that *C. carandas* extract has health beneficial effects and the potential to be used as a natural anti-skin aging ingredient. However, the anti-skin-aging properties of *C. carandas*, especially in terms of matrix metalloproteinases (MMPs), elastase, and hyaluronidase inhibition, have been rarely investigated. Therefore, the present study was the first to reveal the anti-skin-aging ability of *C. carandas* extracts. Additionally, the chemical compositions, cytotoxicity effect on human skin cells, antioxidant, anti-inflammation, and anti-tyrosinase activities of *C. carandas* extracts were also reported.

## 2. Materials and Methods

### 2.1. Plant Materials

Leaves and ripened fruits of *C. carandas* were collected from Samut Songkhram Province, Thailand during May 2017. All plant materials were identified and authenticated by Ms. Wannaree Charoensup, a botanist at the Herbarium of Faculty of Pharmacy, Chiang Mai University. A voucher specimen number 0023269 of *C. carandas* has been deposited in an Herbarium, Department of Pharmaceutical Science, Faculty of Pharmacy, Chiang Mai University. Seed and pulp of *C. carandas* were separated from the fruits. Each part of *C. carandas*, including leaf, seed, and pulp were sliced into small pieces and dried in a hot air oven (UF110, Memert, Germany) set at 50 °C until dryness. The dried *C. carandas* materials were then ground into a fine powder with a Moulinex mixer blender (Moulinex DB81 blender, Paris, France)*.*

### 2.2. Chemical Materials

Ursolic acid, hyaluronidase from bovine testis (E.C. 3.2.1.3.5), MMP-1 from *Clostridium histolyticum* (ChC-E.C. 3.4.23.3), porcine pancreatic elastase (E.C. 3.4.21.36), *N*-[3-(2-furyl) acryloyl]-Leu-Gly-Pro-Ala, *N*-succinyl-Ala-Ala-Ala-*p*-nitroanilide, octylphenol ethoxylate (Triton X-100), alcian blue 8GX, hyaluronic acid, sodium phosphate, sodium chloride, glycerol, and sodium acetate were purchased from Sigma-Aldrich (St. Louis, MO, USA). Protein markers, 30% acrylamide/bis solution, 1 M Tris-HCl, pH 8 solution, and 0.5 M Tris-HCl, pH 6.8 solution were purchased from Bio-Rad Laboratories (Richmond, CA, USA). Sodium dodecyl sulfate (SDS) and bromophenol blue were purchased from Merck (Darmstadt, Germany). Ethanol, hydrochloric acid, ethyl acetate, methanol, *n*-hexane, acetic acid, and dimethyl sulfoxide (DMSO) were purchased from Labscan (Dublin, Ireland). HPLC grade ethanol was purchased from Carlo Erba Reagents (Cornaredo, Italy). Folin–Ciocalteu solution was purchased from Sigma-Aldrich (St. Louis, MO, USA). Gallic acid, quercetin, Trolox, 2,20-azino-bis-3-ethylbenzthiazoline-6-sulphonic acid (ABTS), 1,1-diphenyl-2-picrylhydrazyl (DPPH), 2,4,6-tri (2-pyridyl)-s-triazine (TPTZ) were purchased from Sigma-Aldrich (St. Louis, MO, USA), sodium carbonate, ferric chloride, ferrous sulfate, sodium acetate, aluminum trichloride, and potassium persulfate were purchased from Merck (Darmstadt, Germany). 3-(4,5-Dimethylthiazol-2-yl)-2,5-diphenyltetrazolium bromide (MTT) was purchased from Sigma-Aldrich (St. Louis, MO, USA). RPMI-1640 medium, Dulbecco’s modified Eagle’s medium (DMEM), and penicillin-streptomycin were purchased from GIBCO Invitrogen (Grand Island, NY, USA). Fetal bovine serum (FBS) was obtained from Biochrom AG (Berlin, Germany). ELISA kits were purchased from eBioscience (San Diego, CA, USA).

### 2.3. Preparation of C. carandas Extracts

Fine powder of *C. carandas* fruit, leaf, seed, and pulp were sequentially macerated under continuous stirring by magnetic stirrer (multi-position magnetic stirrer with heater AM4, EOS Scientific Co. Ltd., Bangkok, Thailand) for 24 h at room temperature with n-hexane (3 cycles), ethyl acetate (3 cycles), and 95% ethanol (3 cycles), respectively. The solvent was then removed under vacuum by a rotary evaporator (N-1300S, EYELA, Tokyo, Japan). Finally, twelve *C. carandas* extracts were obtained, i.e., *n*-hexane fruit extract (HF), *n*-hexane leaf extract (HL), *n*-hexane seed extract (HS), *n*-hexane pulp extract (HP), ethyl acetate fruit extract (AF), ethyl acetate leaf extract (AL), ethyl acetate seed extract (AS), ethyl acetate pulp extract (AP), ethanolic fruit extract (EF), ethanolic leaf extract (EL), ethanolic seed extract (ES), and ethanolic pulp extract (EP). *C. carandas* extracts were kept in a tight container and refrigerated (4–8 °C) until further research.

### 2.4. Determination of Ursolic Acid Content by High Performance Liquid Chromatography (HPLC)

Each *C. carandas* extract was analyzed for ursolic acid content using Hitachi HPLC series L (Tokyo, Japan) with a UV detector (Hitachi L-7420, Tokyo, Japan) set at 210 nm. A stationary phase was reverse phase column, Luna 5.0 μ C18 (2) 100A (250 × 2.0 mm, 5 μm) (Phenomenex, Torrance, CA, USA). Mixture of HPLC-grade ethanol and DI water (90:10) was used as an isocratic mobile phase with a flow rate of 0.5 mL/min. All samples were filtrated through 0.2 μm nylon filter (GNWP04700, Millipore, Darmstadt, Germany) prior to the analyzation. The injection volume of each sample was 20 μL. Ursolic acid in a concentration range of 1–500 μg/mL was used to construct a linear regression equation. The ursolic acid content of each *C. carandas* extracts was calculated using the following equation:

*y* = 3475.3*x* + 5730.5 (*R*^2^ = 0.9997), (1)

where *y* was area under the curve (AUC) of ursolic acid peak which was detected around 9.3 min and *x* was a concentration of ursolic acid (g/mg extract). Every experiment was carried out in triplicate.

### 2.5. Determination of Total Phenolic Content of C. carandas Extracts by Folin–Ciocalteu Method

The total phenolic content of each *C. carandas* extract was evaluated using Folin–Ciocalteu method regarding to Chaiyana et al. [10], which was modified from the method of Li et al. [11]. The results were presented as gallic acid equivalent (GAE), which was milligram gallic acid equivalent per gram of each *C. carandas* extracts. Every experiment was carried out in triplicate.

### 2.6. Determination of Total Flavonoid Content of C. carandas Extracts by Dowd Method

The flavonoid content of each *C. carandas* extract was determined using Dowd method according to [12]. The results were reported as quercetin equivalent (QE), which was milligram quercetin equivalent per gram of each *C. carandas* extracts. Every experiment was carried out in triplicate.

### 2.7. Determination of Antioxidant Activities of C. carandas Extracts

*C. carandas* extracts were investigated for antioxidant activities using ferric reducing antioxidant power (FRAP) assay, 2,2′-azino-bis-3-ethylbenzthiazoline-6-sulphonic acid (ABTS) assay, and 1,1-diphenyl-2-picrylhydrazyl (DPPH) assay. Every experiment was carried out in triplicate.

#### 2.7.1. Ferric Reducing Antioxidant Power (FRAP) Assay

The ferric reducing antioxidant power of each *C. carandas* extract was evaluated using the FRAP assay described by Chaiyana et al. [10], which was modified from Benzie and Strain’s method [13]. The absorbance was determined at 590 nm by a microplate reader Sp (220–1000 nm) (SPECTROstar Nano, BMG Labtech, Offenburg, Germany). Ferrous sulfate was also investigated and the resulting data were used to establish a standard curve. The results of reducing power were expressed as equivalent concentration (EC_1_) which represented the concentration of ferrous sulfate (mM) that exhibited an ability to reduce ferric ions (Fe^3+^) to ferrous ion (Fe^2+^) equivalent per g of *C. carandas* extracts.

#### 2.7.2. 2,2′-Azino-Bis-3-Ethylbenzthiazoline-6-Sulphonic Acid (ABTS) Assay

The radical scavenging potential of each *C. carandas* extract was determined using an ABTS assay, as previously defined by Chaiyana et al. [10], which was adapted from Pellegrini et al. [14]. The absorbance was determined at 750 nm by a microplate reader Sp (220–1000 nm) (SPECTROstar Nano, BMG Labtech, Offenburg, Germany). Trolox was also analyzed and used for construction of a standard curve. The results were presented as a Trolox equivalent antioxidant capacity (TEAC) which represented mM concentration of Trolox solution equivalent per g of *C. carandas* extracts.

#### 2.7.3. 1,1-Diphenyl-2-Picrylhydrazyl (DPPH) Assay

The radical scavenging ability of each *C. carandas* extract was determined using the DPPH assay Chaiyana et al. [10], which was adapted from the method defined by Blois [15]. The absorbance was determined at 520 nm by a microplate reader Sp (220–1000 nm) (SPECTROstar Nano, BMG Labtech, Offenburg, Germany). The scavenging activity of each extract was calculated from the following equation:

DPPH inhibition (%) = {1 − [(*A* − *B*)/(*C* − *D*)]} × 100, (2)

where *A* is an UV absorbance of DPPH reagent with sample solution, *B* is an UV absorbance of the sample solution without DPPH reagent, *C* is an UV absorbance of DPPH reagent without sample solution, and *D* is an UV absorbance of the native solvents.

### 2.8. Determination of Anti-Aging Activities of C. carandas Extracts

*C. carandas* extracts were investigated for anti-aging activities via the determination of MMP-1, MMP-2, MMP-9, elastase, and hyaluronidase inhibition. Every experiment was carried out in triplicate.

#### 2.8.1. Determination of MMP-1 Inhibitory Activity by Spectrophotometric Method

The MMP-1 inhibitory activity of each *C. carandas* extract was evaluated using spectrophotometric methods adapted from Chaiyana et al. [16] and Thring et al. [17]. The inhibitory behavior against MMP-1 was calculated from the equation:

MMP-1 inhibition (%) = (1 − *A/B*) × 100, (3)

where *A* and *B* are the UV absorbance of the mixture with and without test sample, respectively.

#### 2.8.2. Determination of MMP-2 and MMP-9 Inhibition by Sodium Dodecyl Sulfate-Polyacrylamide Gel Electrophoresis (SDS-PAGE)

The MMP-2 and MMP-9 inhibitory activity of each *C. carandas* extract was evaluated using SDS-PAGE as previously stated in the study of Chaiyana et al. [16]. Among various subclones of Albino Swiss Mouse Embryo Fibroblasts (3T3), NIH/3T3 cell line, which expressed MMP genes [18], was used in the present study. NIH-3T3 cell line was commonly used instead of skin cells because it exhibited comparable results [19]. Besides, fibroblasts are present throughout the matrix and connective tissue of the body. Therefore, NIH-3T3 cell line has been previously used for studying the regulation of fibrillar collagen synthesis, cellular cytotoxicity, genotoxicity, inhibition of UV-induced skin photoaging, inhibition of MMPs, etc., of tested compounds or topical formulations [20,21,22,23,24,25,26,27,28]. Briefly, NIH/3T3 cell line were cultured in DMEM culture medium with 10.0% newborn calf serum, and 1.0% antibiotic–antimycotic (100×). Cells were incubated in a humidified incubator with a condition of 95.0% air and 5.0% CO_2_ at 37.0 °C. All medium in the culture flask (2.0 mL) was removed and replaced with an equal volume of fresh medium every day. On day 7 to day 10 of culture, the medium in the culture flask was collected and then replaced with equal volume of fresh medium. The collected medium was kept in the freezer for further study.

The SDS-PAGE was then performed using a method of Chaiyana et al. [16]. MMP-2 and MMP-9 expression levels were calculated using the Quality One 1-D Analysis program (Biorad, CA, USA). The inhibitions against MMPs were calculated from the equation:

MMPs inhibition (%) = (1 − *A/B*) × 100, (4)

where *A* and *B* are the MMPs expression of NIH/3T3 cells treated with and without sample, respectively.

#### 2.8.3. Determination of Elastase Inhibitory Activity by Spectrophotometric Method

The inhibitory activity of each *C. carandas* extract against elastase was determined using a spectrophotometric approach adapted from Chaiyana et al. [16] and Thring et al. [17]. The inhibitory activity against elastase was then calculated from the equation:

Elastase inhibition (%) = (1 − *A/B*) × 100, (5)

where *A* and *B* are the UV absorbance of the mixture with and without test sample, respectively.

#### 2.8.4. Determination of Hyaluronidase Inhibitory Activity by Sodium Dodecyl Sulfate-Polyacrylamide Gel Electrophoresis (SDS-PAGE)

The SDS-PAGE was carried out in accordance with a method previously defined by Chaiyana et al. [16]. Briefly, hyaluronidase was incubated with or without the extract at 37 °C for 48 h. The resulting mixture was then mixed with loading dye and loaded into each well of SDS-polyacrylamide gel containing 0.17% hyaluronic acid. After applying the voltage, the bands gently moved down and hyaluronidase was separated. Along with the enzyme separation, the digested hyaluronic acid by hyaluronidase was removed from the gel. Then the gel was washed, incubated in reaction buffer pH 5.0 for 16 h, soaked in staining buffer of Alcian blue for 1 h, and incubated in de-staining buffer until the protein marker showed up. Since hyaluronidase digested hyaluronic acid in the gel, the results would be detected as a clear area. In contrast, if a sample exhibited anti-hyaluronidase activity, its substrate would not be digested, and no clear area detected. The hyaluronidase expression was determined using the Quality One 1-D Analysis program (Biorad, CA, USA). The inhibitions against hyaluronidase were calculated from the equation:

Hyaluronidase inhibition (%) = (1 − *A/B*) × 100, (6)

where *A* and *B* are the hyaluronic acid expression after treated with sample and without sample, respectively.

### 2.9. Anti-Inflammatory Activity Determination

*C. carandas* extracts were investigated for anti-inflammatory activities via the determination of NF-κB, IL-6, and TNF-α inhibition. Every experiment was carried out in triplicate.

#### 2.9.1. Determination of NF-κB Expression by Western Blot Analysis

Firstly, the cell viability of U937 cells (American Type Culture Collection, VA, USA) was investigated using the MTT colorimetric method, which was slightly modified from the previous analysis of Anuchapreeda et al. [29]. U937 cell viability was calculated from the equation:

Cell viability (%) = (1 − *A/B*) × 100, (7)

where *A* and *B* are the optical density of vehicle control and test sample, respectively. The concentration of each *C. carandas* extract showing 80% of cell viability was selected for the further immunoblotting study. Every experiment was carried out in triplicate. The level of NF-κB expression was measured using Western blotting, as defined previously by Chaiyana et al. [30]. Indomethacin, generally known as nonsteroidal anti-inflammatory drug (NSAID), was used as a positive control.

#### 2.9.2. Determination of IL-6 and TNF-α Secretion by Enzyme-Linked Immunosorbent Assay (ELISA)

IL-6 and TNF-α secretion was determined by ELISA according to the method of Chaiyana et al. [16]. Dexamethasone, generally known as a steroid used for anti-inflammation, was used as a positive control. On the other hand, negative control was the LPS-induced RAW 264.7 cells (American Type Culture Collection, VA, USA) treated without sample and vehicle control was the RAW 264.7 cells treated with nothing. The cell viability of RAW 264.7 cells was measured using the MTT colorimetric technique, which was modified from the methods previously defined by Chaiyana et al. [16] and Mueller et al. [31]. RAW 264.7 cells viability was then calculated from the equation:

Cell viability (%) = (1 − *A/B*) × 100, (8)

where *A* is an optical density of vehicle control and *B* is an optical density of sample, positive control, or negative control. To limit variability due to cell density differences, IL-6 and TNF-secretions were standardized to MTT values [31]. The secretions of those cytokines from negative control, which were RAW 264.7 cells treated with only LPS, was described as 100%. Additionally, % inhibition against IL-6 and TNF-α was calculated by subtracting the cytokines secretion from 100%.

### 2.10. Anti-Tyrosinase Determination by Dopachrome Method

The anti-tyrosinase activity of each *C. carandas* extract was determined using DOPAchrome method regarding to a method of Momtaz et al. [32] which was adapted from Curto et al. [33] and Nerya et al. [34]. As substrates, L-tyrosine and L-DOPA were used. As a positive control, kojic acid was used. The inhibitory activity against tyrosinase was calculated from the equation:

Tyrosinase inhibition (%) = (1 − *A/B*) × 100, (9)

where *A* and *B* are the absorbance of the mixture with and without test sample, respectively. Every experiment was carried out in triplicate.

### 2.11. Cytotoxicity of C. carandas Extracts in Human Keratinocyte (Hacat) Cells by MTT Colorimetric Technique

The cell viability of sample-treated HaCaT cells (American Type Culture Collection, VA, USA) was calculated using the MTT colorimetric procedure, which was modified from the methods of Colombo et al. [35] and Anuchapreeda et al. [29]. The viability of HaCaT cells was investigated using the MTT colorimetric method. HaCaT cell viability was calculated from the equation:

Cell viability (%) = (1 − *A/B*) × 100, (10)

where *A* and *B* are the optical density of vehicle control and test sample, respectively. The concentration of each *C. carandas* extract showing 80% of cell viability was selected for the further immunoblotting study. Every experiment was carried out in triplicate.

### 2.12. Statistical Analysis

All data were provided in the form of a mean ± standard deviation (S.D.). Individual differences were assessed using one-way analysis of variance (ANOVA), which was supplemented by Tukey’s post-hoc analysis. * *p* < 0.05, ** *p* < 0.01, and *** *p* < 0.001 indicated statistical significance.

## 3. Results and Discussion

### 3.1. C. carandas Extracts

**Figure 1:**
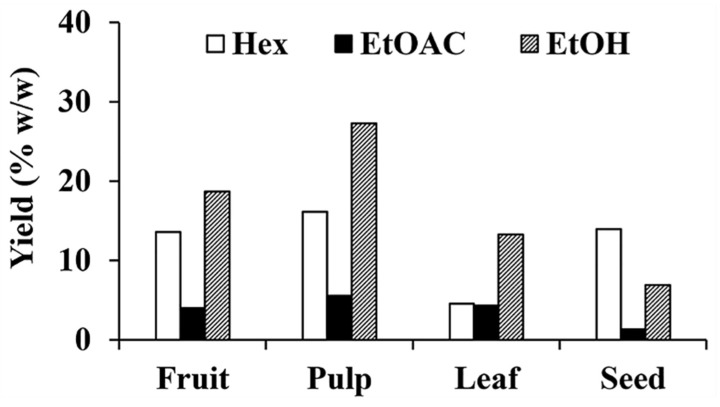
Yields of *C. carandas* extracts sequentially extracted using *n*-hexane (Hex), ethyl acetate (EtOAc), and 95% ethanol (EtOH), respectively.

The yields of each *C. carandas* extracts are shown in Figure 1. *n*-Hexane and ethanolic extracts were a semisolid mass. On the other hand, all ethyl acetate extracts were solid powder. The color of each extract depended on the *C. carandas* materials, e.g., the extracts from the leaf, seed, pulp, and fruit part had a dark green, yellow–brown, dark purple, and dark brown color, respectively. Interestingly, ethanolic extracts showed the significantly highest yields in fruit, pulp, and leaf part of *C. carandas*, whereas the significantly highest yield of the extracts from seed part was obtained from the *n*-hexane extracts. The likely explanation was due to the high content of fatty acid, i.e., palmitic acid and stearic acid, in the seed part [4] but high content of phenolic compounds in the others [36,37]. Therefore, it was likely that *C. carandas* fruit, pulp, and leaf contained polar phytoconstituents which were extracted well by polar solvent. In contrast, the *C. carandas* seed contained nonpolar phytoconstituents which was extracted well by nonpolar solvent.

Among twelve *C. carandas* extracts, PE showed the highest yield which was 27.3% *w*/*w*. Besides, the pulp part yielded the highest extract amount in all extracted solvent used. The likely explanation was because an edible pulp is rich with several bioactive compounds and mineral contents.

### 3.2. Ursolic Acid Content of C. carandas Extracts

**Figure 2:**
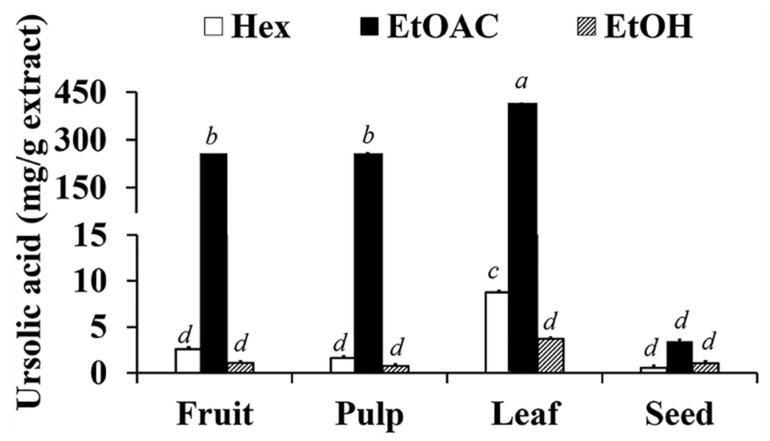
Ursolic acid content of *C. carandas* extracts sequentially extracted using *n*-hexane (Hex), ethyl acetate (EtOAc), and 95% ethanol (EtOH), respectively. The letter *a*, *b*, *c*, and *d* denote significant difference among different *C. carandas* extracts (*p* < 0.05) after being analyzed by one-way ANOVA followed by Tukey’s test.

The ursolic acid content of *C. carandas* extracts are shown in Figure 2. The results indicated that ethyl acetate was the most suitable solvent for ursolic acid extraction since the ursolic acid contents were significantly highest in all ethyl acetate extracts, including the extract from fruit, leaf, pulp, and seed part (*p* < 0.05). Although the previous ursolic acid solubility study noted that ursolic acid was well dissolved in ethanol (solubility = 16.808 ± 0.824 mg/mL), followed by ethyl acetate (solubility = 6.857 ± 0.359 mg/mL) and *n*-hexane (solubility = 0.471 ± 0.064 mg/mL) [38,39], in this study it was revealed that ethyl acetate extracts contained a significantly higher amount of ursolic acid compared to ethanolic extracts. The reasons were due to a subsequential extraction process. Since the *C. carandas* dried powder was macerated in ethyl acetate before ethanol, ursolic acid was therefore extracted and existed in the ethyl acetate extract, leading to lower ursolic acid content detected in ethanolic extracts.

**Figure 3:**
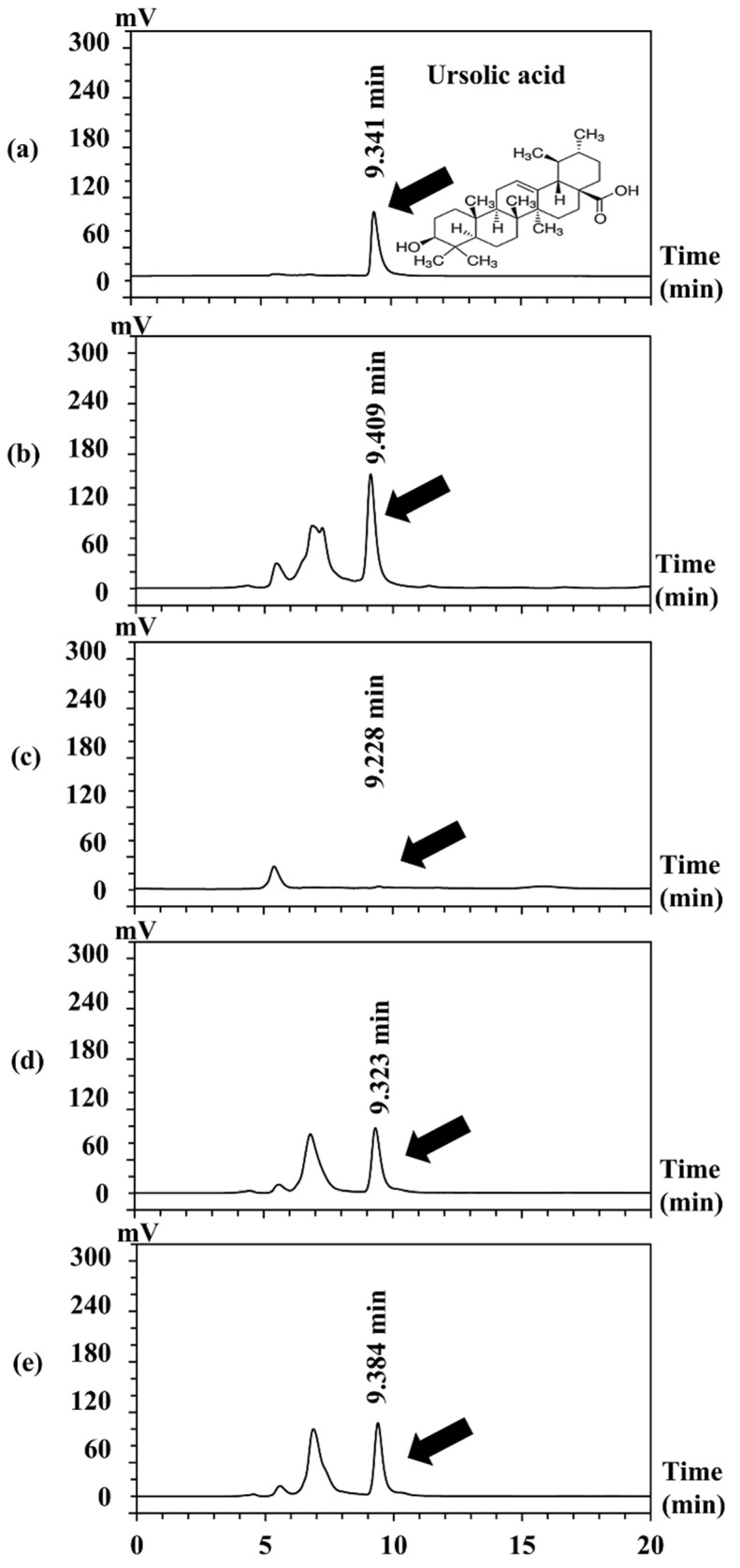
HPLC chromatograms of ursolic acid (**a**) and ethyl acetate extracts from various parts of *C. carandas*, i.e., leaf (**b**), seed (**c**), pulp (**d**), and fruit (**e**).

Among various parts of *C. carandas* extracted using ethyl acetate, AL contained the significantly highest ursolic acid (411.8 mg/g extract), followed by AP (256.34 mg/g extract), AF (253.32 mg/g extract), and AS (3.4 mg/g extract), respectively (*p* < 0.05). The HPLC chromatograms of ethyl acetate extracts from different part of *C. carandas* are shown in Figure 3. The major peaks detected in chromatograms of *C. carandas* ethyl acetate extracts were at the retention time ranging from 9.228 to 9.409 min which were close to that of ursolic acid (9.341 min). Therefore, it could be concluded that ursolic acid was a main abundant compound in *C. carandas* extracts.

On the other hand, among various part of *C. carandas* extracted using *n*-hexane, HL contained the significantly highest ursolic acid (8.77 mg/g extract) (*p* < 0.05), whereas HF (2.54 mg/g extract), HP (1.60 mg/g extract), and HS (0.55 mg/g extract) contained no significantly different ursolic acid content (*p* > 0.05). In contrast, different part of *C. carandas* extracted using ethanol yielded no significant difference in ursolic acid content (*p* > 0.05).

Since ursolic acid has been reported for several pharmacological activities, such as anti-inflammation, anti-tumor, and antibacterial activities [40], it could be used as a marker for further quantitative analysis of *C. carandas* extract.

### 3.3. Total Phenolic and Flavonoid Content of C. carandas Extracts

TPC and TFC of *C. carandas* extracts are shown in Table 1. Among various solvents, ethanolic extracts yielded the significantly highest phenolic and flavonoid content comparing to the others (*p* < 0.05). Therefore, ethanol was noted as the most suitable solvent for extracting phenolics and flavonoids. The likely explanation was due to its hydrophilicity. The previous study reported that the highest TPC was obtained from extraction using methanol (ε = 33.0), followed by ethanol (ε = 25.3) and acetone (ε = 20.7), respectively [41]. Although more polar solvent could extract more phenolic compounds, ethanol was used in the present study and suggested for further study since it is widely known as safe and low cost. Additionally, ethanol has been reported to extract more flavonoids comparing to methanol and acetone [41].

Among ethanolic extracts from different *C. carandas* parts, ES yielded the significantly highest phenolic content with the GAE of 265.1 ± 1.4 mg/g extract, followed by EL (219.7 ± 4.8 mg/g extract), EF (61.2 ± 4.4 mg/g extract), and EP (24.2 ± 1.0 mg/g extract), respectively. Interestingly, the present study is the first to report a remarkable phenolic content in *C. carandas* seed.

On the other hand, EP yielded the significantly highest flavonoid content with the QE of 14.1 ± 0.2 mg/g extract, followed by EF (10.1 ± 0.3 mg/g extract), EL (8.8 ± 2.2 mg/g extract), and ES (6.8 ± 0.1 mg/g extract), respectively. The results noted that other than ursolic acid, triterpenoid, phenolics, and flavonoids were also detected in various parts of *C. carandas*. The results were well accordant with previous studies which reported that various phenolics and flavonoids have been detected in *C. carandas*, especially vanillic acid in fruit, as well as ellagic acid and quercetin in pulp [37,42,43].

### 3.4. Antioxidant Activities of C. carandas Extracts

Ethanolic extract from various part of *C. carandas* possessed the highest antioxidants activity via both scavenging and reducing abilities as shown in Table 1. Among different ethanolic extracts, EL possessed the significantly highest antioxidant activities with EC_1_ of 3.53 ± 0.16 mM/g extract, TEAC value of 496.0 ± 6.2 µM/g extract, and IC_50_ against DPPH^•^ of 16.2 ± 0.1 µg/mL (*p* < 0.05). Additionally, ES also showed comparable ABTS^•+^ and DPPH^•^ scavenging activity to EL with TEAC value of 492.8 ± 4.1 µM/g extract and IC_50_ against DPPH^•^ of 16.0 ± 0.5 µg/mL (*p* > 0.05). The antioxidant results were well accordant with TPC, but not related to TFC and ursolic acid content. Therefore, it could be concluded that phenolics were major compounds responsible for the antioxidant properties of *C. carandas* extracts. Since research has proposed that topical antioxidants be used for the treatment of skin aging [44,45,46], *C. carandas* extracts, especially EL, have been suggested to be used as natural antioxidants, which could prevent skin aging. A previous study reported that antioxidants play an important role in the prevention and treatment of UV-induced skin aging due to the reduction in oxidized proteins, which strongly correlates with the severity of photo-aging clinical characteristics [47].

### 3.5. MPPs Inhibitory Activities of C. carandas Extracts

**Figure 4:**
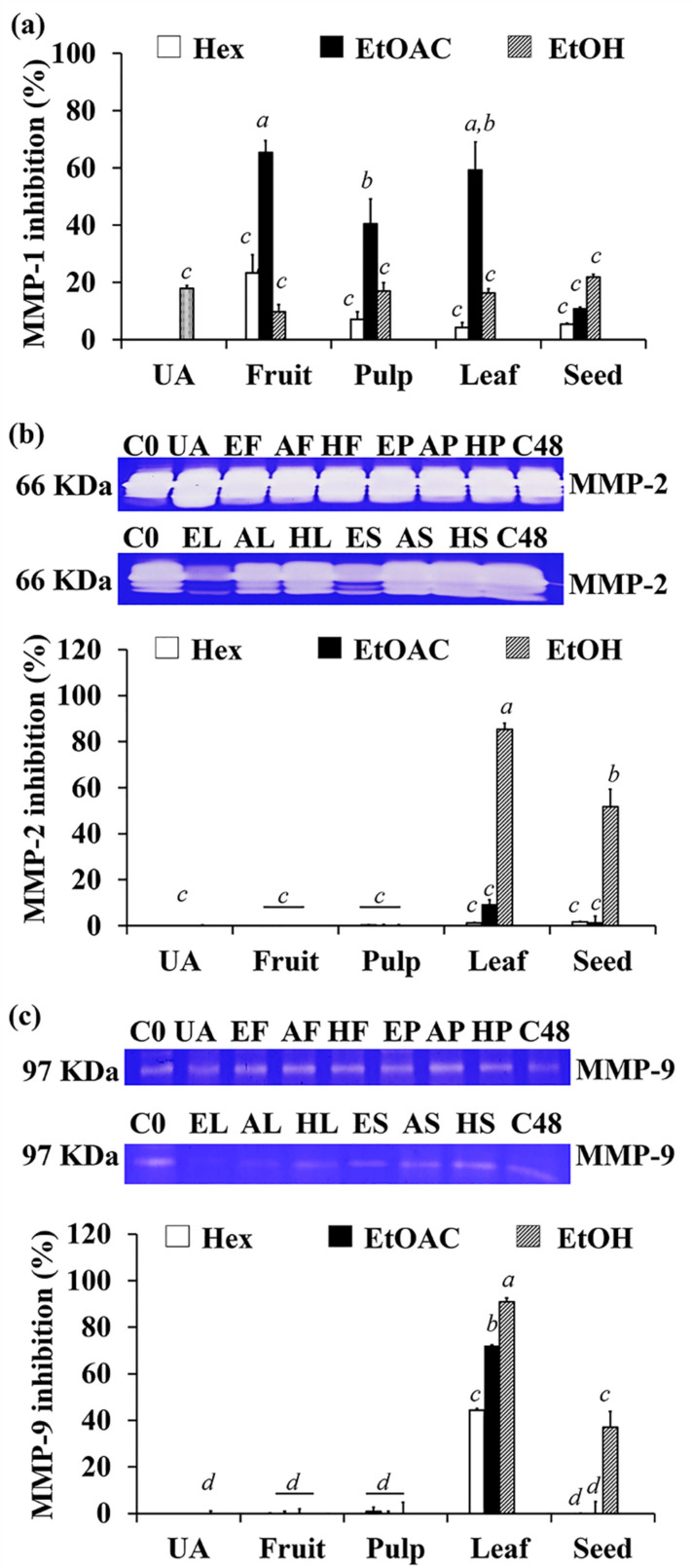
Inhibition against MMP-1 (**a**), MMP-2 (**b**), and MMP-9 (**c**) of 1 mg/mL ursolic acid (UA) and various parts of *C. carandas* sequentially extracted using *n*-hexane (Hex), ethyl acetate (EtOAc), and 95% ethanol (EtOH), respectively. DMSO was used as a vehicle control at 0 h (C0) and 48 h (C48). Various *C. carandas* extracts included *n*-hexane extracts from fruit (HF), leaf (HL), seed (HS), and pulp (HP); ethyl acetate extracts from fruit (AF), leaf (AL), seed (AS)*,* and pulp (AP); ethanolic extracts from fruit (EF), leaf (EL), seed (ES), and pulp (EP). The letter *a*, *b*, *c*, and *d* denote significant difference among different *C. carandas* extracts (*p* < 0.05) after analyzed by one-way ANOVA followed by Tukey’s test. ND refer to not detected value.

MMPs are a form of zinc-dependent endopeptidase that degrades ECM components and is involved in dermal turnover and remodeling [48]. There are several different types of MMPs, but only a few have been associated to skin wrinkles, including collagenases, which break down collagens (MMP-1), and gelatinases, which degrade denatured collagens (MMP-2 and MMP-9) [49]. The inhibitory activities of *C. carandas* extracts against these MMPs are shown in Figure 4. There were multiple bands detected around 66 KDa because MMP-2 could be present in both pro MMP-2 and the active form. Pro MMP-2 in its full-length of 72 kDa could be activated by proteolytic cleavage to an enzymatically active form, which was around 62–67 kDa [50,51,52]. Since the size of pro MMP-2 and its active form were very close, the resultant band was oversaturated and overlapped. However, the results were calculated from the summation of all bands detected around 66 KDa.

Ethyl acetate extracts tended to possess significantly higher MMP-1 inhibition, whereas ethanolic extracts tended to possess significantly higher MMP-2 and MMP-9 inhibition (*p* < 0.05). Among ethyl acetate extracts, AF and AL possessed the significantly highest inhibition against MMP-1 (*p* < 0.05) with the inhibition of 65.4 ± 4.0% and 59.2 ± 9.8%, respectively (*p* < 0.05). On the other hand, EL possessed the significantly highest inhibition against both MMP-2 and MMP-9 with the inhibition of 85.3 ± 2.8% and 90.2 ± 1.6%, respectively (*p* < 0.05). Although the gelatin zymography results of MMP-2 are oversaturated, making it difficult to confirm the exact results, the inhibitions against MMP-2 were distinctly observed in EL and ES. The likely explanation lies in the different hemopexin-like C-terminal domain in MMP-1, MMP-2, and MMP-9 that conducted the specific binding site for substrates and inhibitors since MMPs are composed of several active domains, including the prodomain, catalytic domain, hemopexin domain, and hinge region [16,53,54]. The specific site three-quarters from the N-terminal of the hemopexin domain in MMP-1 plays an important role in cleavage of substrates such as collagens type I, II, and III [53,54], while MMP-2 and MMP-9 have three repeats of a type II fibronectin domain embedded in the catalytic domain, and the groove in the hemopexin domain helps with substrate binding [16,53]. Therefore, it could be summarized that the ethyl acetate extracts (especially AF and AL) were more specific for MMP-1 inhibition, whereas the ethanolic extract (especially EL) were more specific for MMP-2 and MMP-9 inhibition. However, these *C. carandas* extracts were suggested for the prevention of skin aging caused by UV-A irradiation. Since UV-A irradiation up-regulated the levels of MMP-1, MMP-2, and MMP-9, which are crucial in the breakdown of almost all extracellular matrix components and result in the decomposition of dermal structures and finally skin aging [55].

### 3.6. Elastase Inhibitory Activities of C. carandas Extracts

**Figure 5:**
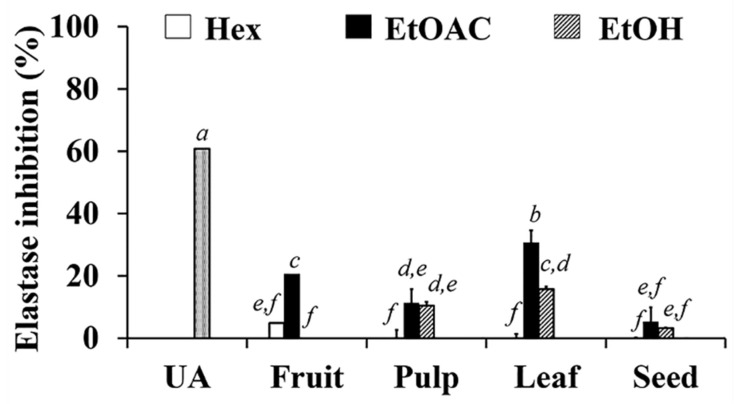
Inhibition against elastase of 1 mg/mL ursolic acid (UA) and various parts of *C. carandas* sequentially extracted using *n*-hexane (Hex), ethyl acetate (EtOAc), and 95% ethanol (EtOH), respectively. DMSO was used as a vehicle control. The letter *a*, *b*, *c*, *d*, *e*, and *f* denote significant difference among different *C. carandas* extracts (*p* < 0.05) after analyzed by one-way ANOVA followed by Tukey’s test.

Elastase degrades elastic fibers, which are mostly found in connective tissue and make up the extracellular matrix that gives skin its elasticity; thus, elastase degeneration is linked to the formation of wrinkles [56,57]. The elastase inhibition of *C. carandas* extracts are shown in Figure 5. Ethyl acetate extracts, particularly the ethyl acetate extract from leaves, were found to be the most effective elastase inhibitors (*p* < 0.05). Ursolic acid was identified as a compound responsible for the elastase inhibitory activity since it possessed a strong inhibition of 60.8 ± 4.0%. Furthermore, the elastase inhibition was greatest in EL, which had the highest ursolic acid content. The findings were consistent with prior research that found ursolic acid inhibited human and porcine leucocyte elastase by binding to the enzyme’s subsites S3–S5 [58,59]. As a result, EL could be a promising active compound for preventing elastase degradation and improving skin elasticity.

### 3.7. Hyaluronidase Inhibitory Activity of C. carandas Extracts

Hyaluronidase is a key hydrolysis enzyme that cleaves the 1,4-glycosidic bond in hyaluronan, of which 50% is found in skin tissue [60]. Because of its high hydration capacity, hyaluronan, which is produced by epidermal keratinocytes and dermal fibroblasts, contributes to the skin’s viscoelastic properties [61,62]. Therefore, hyaluronan degradation in the skin by hyaluronidase resulted in a loss of skin moisture and laxity, which are all signs of aging skin. Compounds that inhibited hyaluronan turnover by inhibiting hyaluronidase activity were helpful for skin rejuvenation.

**Figure 6:**
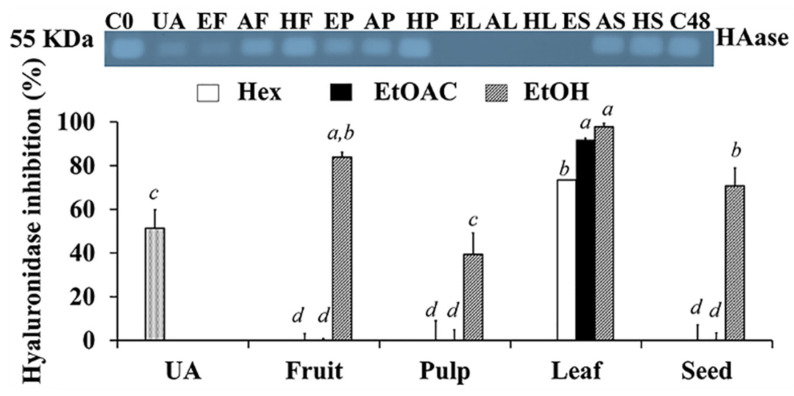
Inhibition against hyaluronidase of 1 mg/mL ursolic acid (UA) and various parts of *C. carandas* sequentially extracted using *n*-hexane (Hex), ethyl acetate (EtOAc), and 95% ethanol (EtOH), respectively. DMSO was used as a vehicle control at 0 h (C0) and 48h (C48). Various *C. carandas* extracts included *n*-hexane extracts from fruit (HF), leaf (HL), seed (HS), and pulp (HP); ethyl acetate extracts from fruit (AF), leaf (AL), seed (AS), and pulp (AP); ethanolic extracts from fruit (EF), leaf (EL), seed (ES), and pulp (EP). The letters *a*, *b*, *c*, and *d* denote significant difference among different *C. carandas* extracts (*p* < 0.05) after being analyzed by one-way ANOVA followed by Tukey’s test.

The inhibitory activity against hyaluronidase of *C. carandas* extracts are shown in Figure 6. The ethanolic *C. carandas* extracts exhibited outstanding hyaluronidase inhibition, particularly from leaves (97.8 ± 1.6%), fruit (83.9 ± 2.2%), and seed (70.7 ± 8.2%), all of which were higher than ursolic acid (51.3 ± 8.5%). The findings were consistent with our previous research, which found that ursolic acid could inhibit hyaluronidase with a 40–60% inhibition at a concentration of 1 mg/mL [16]. Therefore, ursolic acid might not be the only compound in *C. carandas* that is responsible for the hyaluronidase inhibition. Other phenolics and flavonoids contained in *C. carandas* may be compounds that inhibit hyaluronidase, as the ethanolic extracts with high levels of phenolics and flavonoids significantly inhibited the enzyme hyaluronidase. The previous structure–activity studies reported that flavonoids, such as luteolin, apigenin, and kaempferol were found to have high inhibitory activity against hyaluronidase, while silybin, myricetin, and quercetin were found to have moderate inhibitory activity [63,64]. Therefore, phenolic and flavonoids from *C. carandas* extracts would be the compounds for inhibiting hyaluronidase.

### 3.8. Anti-Inflammatory Activity of C. carandas Extracts

Inflammation underlies a broad range of pathological and physiological processes [65]. NF-κB is released and translocated to the nucleus after the phosphorylation of the inhibitory IκB, and consequently regulates the expression of many genes involved in inflammatory signaling, cell proliferation, and cell apoptosis. Because the nuclear factor NF-κB signaling pathway has long been considered to be a model of proinflammatory signaling, NF-κB inhibitors may be useful as anti-inflammatory agents [66]. In addition, the inflammatory cytokines (TNF-α, IL-6, and others), which are primarily produced by macrophages and mast cells, play a number of functions in the inflammatory response, including stimulating endothelial cells and leukocytes, as well as triggering the acute-phase response [65].

**Figure 7:**
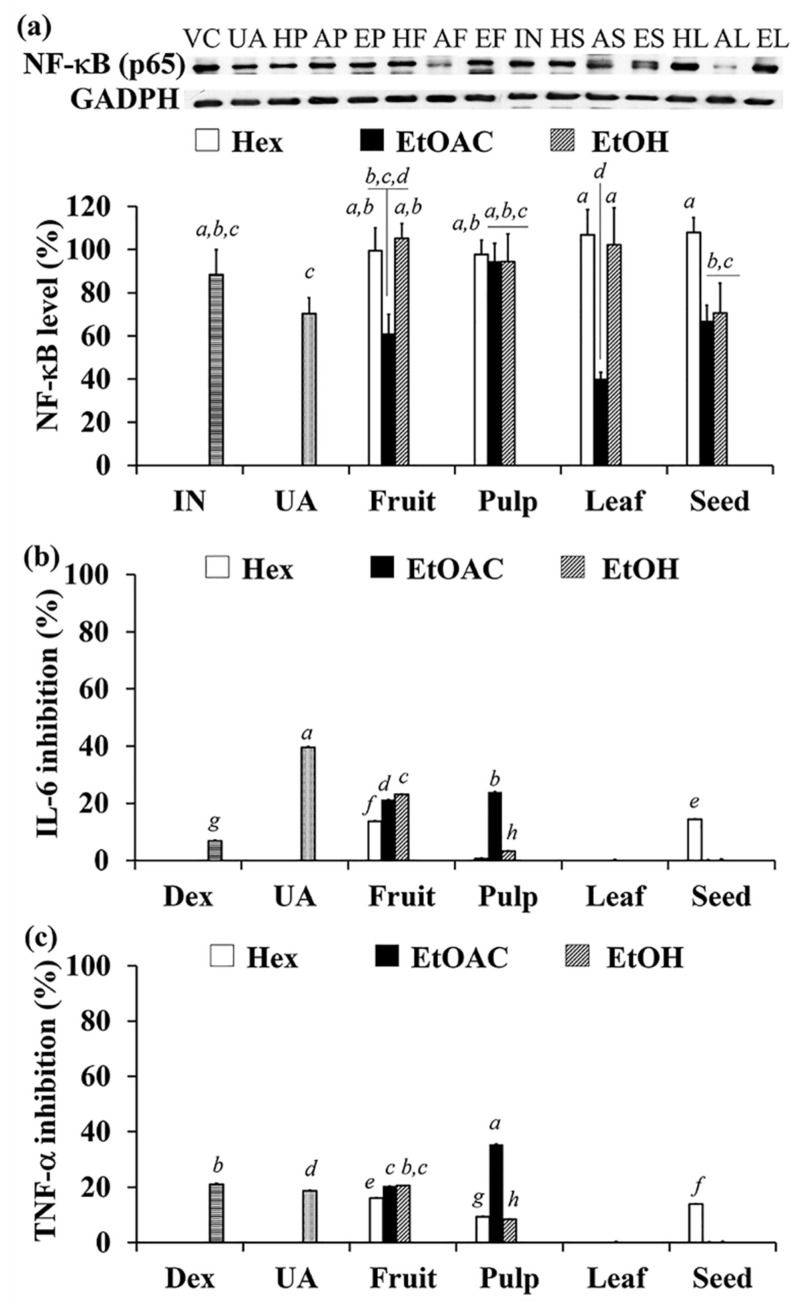
The effect of ursolic acid (UA), and *C. carandas* sequentially extracted using *n*-hexane (Hex), ethyl acetate (EtOAc), and 95% ethanol (EtOH), respectively, on NF-κB protein expressions in U937 cells (**a**), IL-6 (**b**) and TNF-α (**c**) secretion in RAW 264.7 cells. Various *C. carandas* extracts included *n*-hexane extracts from fruit (HF), leaf (HL), seed (HS), and pulp (HP); ethyl acetate extracts from fruit (AF), leaf (AL), seed (AS), and pulp (AP); ethanolic extracts from fruit (EF), leaf (EL), seed (ES), and pulp (EP). Dimethyl sulfoxide was used as vehicle control (VC). Indomethacin (IN) and dexamethasone (Dex) were used as reference standard. The letters *a*, *b*, *c*, *d*, *e, f*, *g*, and *h* denote significant difference among different *C. carandas* extracts (*p* < 0.05) after being analyzed by one-way ANOVA followed by Tukey’s test.

The anti-inflammatory activities of *C. carandas* extracts indicated that several *C. carandas* extracts displayed potent anti-inflammatory activities, as shown in Figure 7. The IC_20_ values (Table 2) represented the concentrations at 80% of viable U937 or RAW 264.7 cells. Each *C. carandas* extract at IC_20_ value was used in the assessment of anti-inflammatory activity. n-Hexane extracts were the safest extracts since they exhibited the highest value of IC_20_ which were greater than 250 µg/mL in U937 cells and 100 µg/mL in RAW 264.7 cells. Except for the leaf part, ethyl acetate extracts had greater effects on U937 and RAW 264.7 cells viabilities than ethanolic extracts.

AL was significantly the most effective inhibiting NF-κB expression with the inhibition of 59.9 ± 3%, which was more potent than ursolic acid (29.7 ± 7.3% inhibition) and indomethacin, a well-known nonsteroidal anti-inflammatory drug (11.6 ± 11.6% inhibition) (*p* < 0.05). This suggests that *C. carandas* extracts contain other compounds that suppress NF-κB protein expression in addition to ursolic acid. In contrast, AP showed the highest inhibitory effects on IL-6 and TNF-α secretions with inhibitory values of 23.9 ± 0.2% and 35.4 ± 0.2%, respectively, which were significantly more potent than dexamethasone (*p* < 0.05) at the same concentration (10 µg/mL). Ursolic acid, a major component of *C. carandas*, was shown to be responsible for IL-6 and TNF-α inhibition, as it had a stronger inhibitory effect than dexamethasone.

The finding from this study remarked that the extracts with the most potent inhibitory activity on NF-κB had no effect on IL-6 or TNF-α secretion inhibition, while the extract with the most potent inhibitory activity on IL-6 and TNF-α secretion had no effect on NF-κB suppression. Although the NF-κB pathway is one of the intracellular signaling pathways to activate IL-6 and TNF-α, there are varieties of mechanisms that would also be involved, such as mammalian mitogen-activated protein kinase (MAPK), Janus kinase 2/signal transducers and activators of transcription 3(JAK2/STAT3), etc. [67,68,69]. Inflammation can be resolved through many mechanisms. In this study, NF-κB and IL-6 were examined in different cell lines (U937 and Raw 264.7 cells, respectively). Thus, it is possible that the result did not relate to each other. NF-IL6 (C/EBP) and NF-κB are proteins involved in IL-6 gene expression in U937 cells [70]. However, Raw 264.7 showed a different signaling pathway that associates with NF-κB and ERK (MAPK) [71,72].

### 3.9. Anti-Tyrosinase Activity of C. carandas Extracts

**Figure 8:**
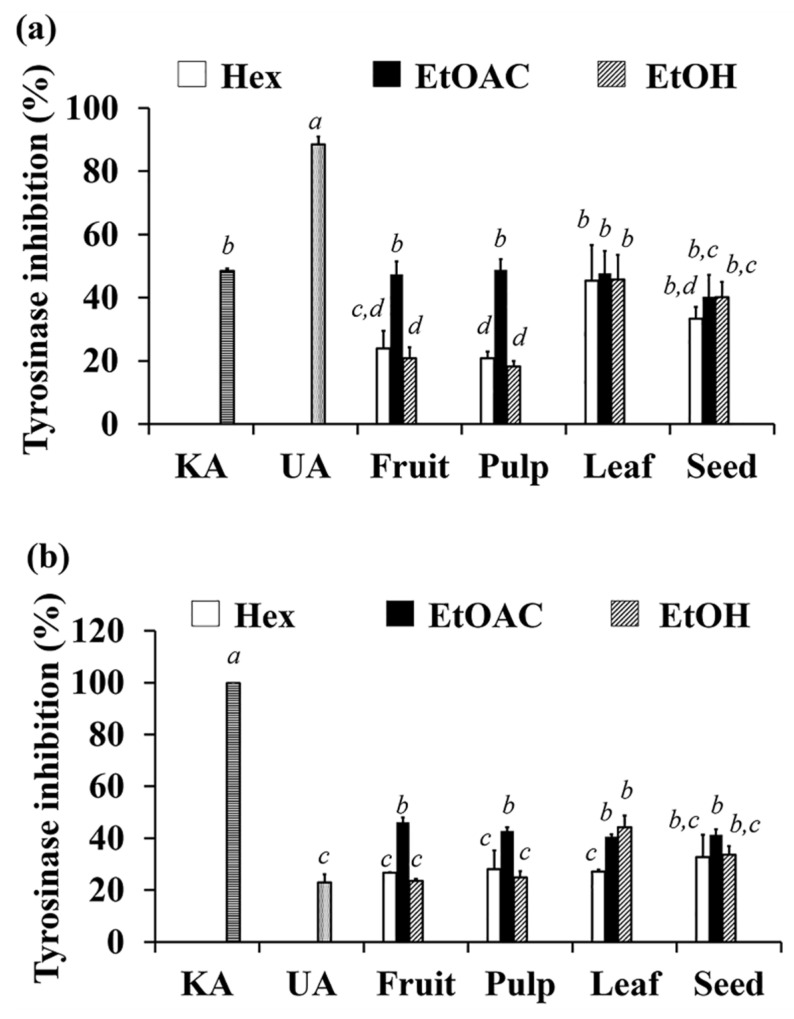
Inhibition against diphenolase (**a**) and monophenolase (**b**) activities of tyrosinase at concentration of 125 µg/mL kojic acid (KA), ursolic acid (UA) and *C. carandas* extracts sequentially extracted using *n*-hexane (Hex), ethyl acetate (EtOAc), and 95% ethanol (EtOH), respectively. The letters *a*, *b* and *c* denote significant difference among different *C. carandas* extracts (*p* < 0.05) after being analyzed by one-way ANOVA followed by Tukey’s test.

The anti-tyrosinase activities of *C. carandas* extracts are shown in Figure 8. The diphenolase (Figure 8a) and monophenolase (Figure 8b) activities of mushroom tyrosinase were evaluated using L-DOPA and L-tyrosine as substrates, respectively.

The significantly greatest inhibitory activity against tyrosinase was found in ethyl acetate extracts from different parts of *C. carandas* (*p* < 0.05). AF (47.3 ± 4.2%), AP (48.9 ± 3.3%), AL (47.7 ± 7.1%), and AS (40.3 ± 6.9%) were effective diphenolase inhibitors with anti-tyrosinase activity not significantly different from that of kojic acid (48.4 ± 0.4%). Ursolic acid was a key component in their anti-tyrosinase efforts, as it exhibited the highest inhibition of 88.5 ± 2.4% (*p* < 0.05).

Similarly, AF (46.2 ± 1.8%), AP (42.9 ± 1.2%), AL (40.5 ± 1.0%), and AS (41.2 ± 2.0%), were the most effective on monophenolase inhibition. But their monophenolase inhibition were higher than that of ursolic acid (22.9 ± 3.1%). It would be explainable that other compounds than ursolic acid may have been responsible for the monophenolase inhibition. Although ursolic acid was more selective to diphenolase than monophenolase, all ethyl acetate extracts were effective on both diphenolase and monophenolase. Therefore, another benefit of natural extract was the synergistic effects of many components contained in the extract, which made them more potent at the same concentration as the pure compound.

### 3.10. HaCaT Cell Cytotoxicity

**Figure 9:**
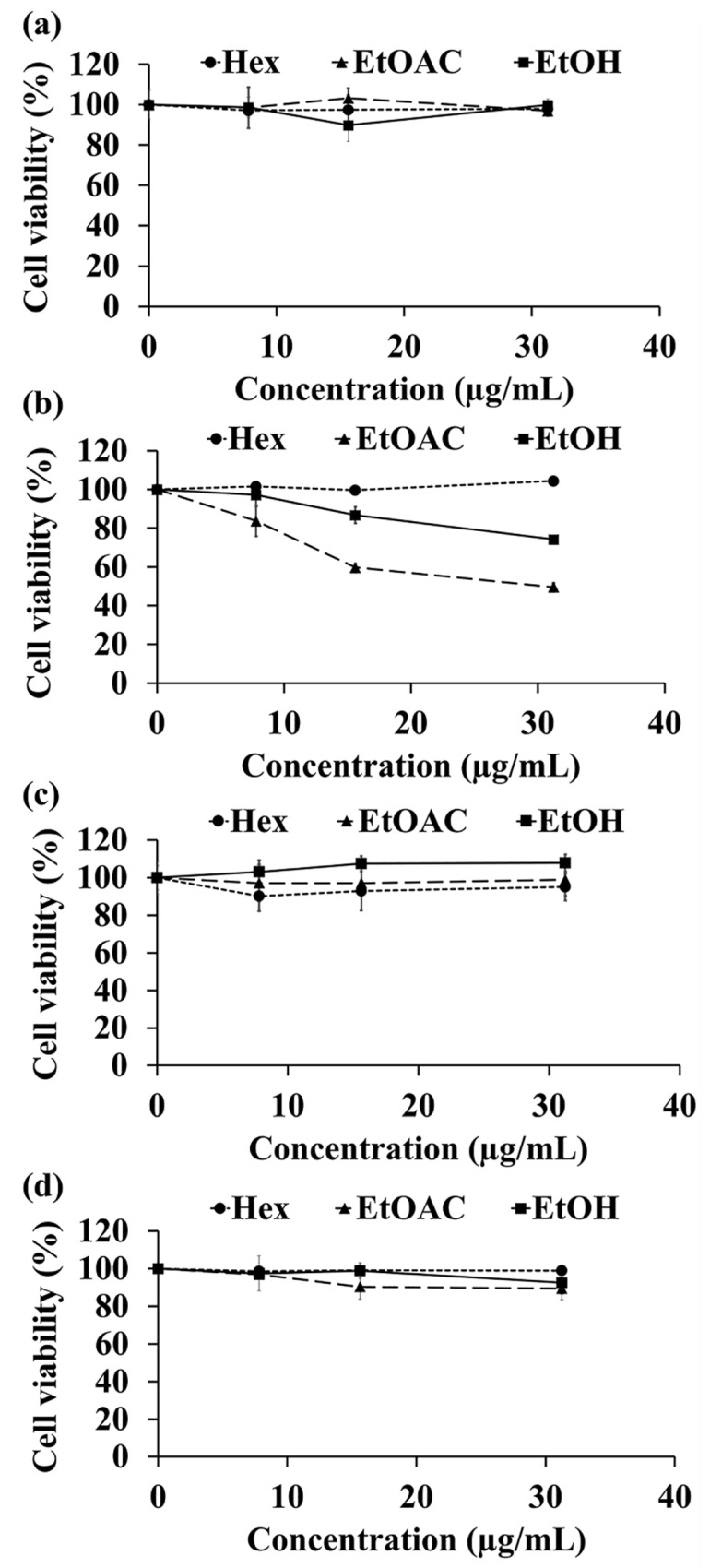
Effect of *C. carandas* extracts from leaf (**a**), seed (**b**), pulp (**c**), and fruit (**d**) which were sequentially extracted using *n*-hexane (Hex), ethyl acetate (EtOAc), and 95% ethanol (EtOH), respectively, on viability of HaCaT after treatment for 48 h. The cell viability of HaCaT was determined by MTT colorimetric technique.

The cytotoxic effect of *C. carandas* extracts at various concentrations ranging from 0 to 31.25 µg/mL on HaCaT cell line is shown in Figure 9. Except for the extracts from the seed, which were SL and SE, none of the *C. carandas* extracts inhibited HaCAT cell growth. It would be concluded that leaf, pulp, and fruit of *C. carandas* had no cytotoxic effect on HaCaT cells.

## 4. Conclusions

Ethyl acetate was able to extract the most ursolic acid from different parts of *C. carandas*, and AL, which had the highest ursolic acid content (411.8 mg/g extract), inhibited MMP-1 activity, NF-κB expression, and tyrosinase activity the most. In contrast, ethanol could extract the greatest amount of phenolics and flavonoids from *C. carandas.* ES, which yielded the significantly highest phenolic content (GAE = 265.1 ± 1.4 mg/g extract), possessed the significantly highest antioxidant activities with TEAC value of 0.49 ± 0.01 mM/g extract and IC_50_ against DPPH^•^ of 16.0 ± 0.5 µg/mL (*p* > 0.05). Furthermore, while EL and ES had comparable ABTS^•+^ and DPPH^•^ scavenging activity, EL had a higher ferric reducing antioxidant power, with an EC_1_ of 3.53 ± 0.16 mM/g extract. Inhibition of MMP-2, MMP-9, and elastase was discovered to be the significantly highest in EL. The findings from this study concluded that *C. carandas* leaves contained the highest level of ursolic acid, phenolics, and flavonoids. However, the solvent used in the extraction process had a significant impact on the chemical compositions and biological activities of *C. carandas* extracts. Although several *C. carandas* extracts, such as AL and EL, have biological activities that are beneficial to human skin, AL has been proposed for use as an active ingredient in cosmetics and cosmeceuticals because of its antiwrinkle, anti-inflammation, and whitening properties.

## Figures and Tables

**Table 1:** Total phenolics content, total flavonoids content, and antioxidant activities of each *C. carandas* extracts.

Samples		GAE (mg Gallic Acid/g Sample)	QE (mg Guercetin/g Sample)	Antioxidant Activities		
				EC_1_ (µM FeSO_4_/g Extract)	TEAC (µM Trolox/g Extract)	IC_50_ on DPPH^•+^ (µg/mL)
Ursolic acid		ND	ND	0.0 ± 0.0 ^f^	5.1 ± 2.1 ^e^	>125.0 ^e^
Gallic acid		ND	ND	22,873 ± 203 ^a^	25.0 ± 2.6 ^c^	1.0 ± 0.0 ^d^
Quercetin		ND	ND	81,461 ± 308 ^b^	58.1 ± 2.7 ^a^	4.4 ± 0.4 ^d^
	Hex	0.5 ± 4.4 ^e^	1.7 ± 0.1 ^e^	0.0 ± 11.6 ^f^	15.1 ± 2.1 ^e^	>125.0 ^e^
Fruits	EtOAc	19.8 ± 3.6 ^d^	4.1 ± 0.5 ^d^	959.5 ± 10.8 ^f^	85.5 ± 7.4 ^d^	420.8 ± 57.4 ^a^
	EtOH	61.2 ± 4.4 ^c^	10.1 ± 0.3 ^b^	470.0 ± 48.7 ^e^	251.7 ± 13.1 ^c^	414.5 ± 20.0 ^a,b^
	Hex	0.2 ± 3.6 ^e^	1.2 ± 0.0 ^e^	0.0 ± 5.5 ^f^	17.2 ± 8.8 ^e^	>125.0 ^e^
Pulp	EtOAc	22.6 ± 2.3 ^d^	2.5 ± 0.1 ^e^	140.9 ± 10.5 ^f^	103.6 ± 9.8 ^d^	312.3 ± 45.7 ^a–c^
	EtOH	24.2 ± 1.0 ^d^	14.1 ± 0.2 ^a^	198.5 ± 5.6 ^e,f^	131.8 ± 3.9 ^d^	224.7 ± 27.8 ^b,c^
	Hex	6.0 ± 2.2 ^e^	4.2 ± 0.2 ^d^	0.0 ± 5.6 ^f^	42.2 ± 27.1 ^e^	>125.0 ^e^
Leaf	EtOAc	18.3 ± 2.0 ^d^	4.8 ± 0.2 ^d^	165.6 ± 14.2 ^f^	123.5 ± 6.5 ^d^	301.8 ± 45.6 ^b^
	EtOH	219.7 ± 4.8 ^b^	8.8 ± 2.2 ^b^	3,531 ± 159 ^c^	496.0 ± 6.2 ^b^	16.4 ± 0.1 ^d^
	Hex	0.0 ± 0.7 ^e^	1.0 ± 0.1 ^e^	35.0 ± 1.9 ^f^	22.2 ± 4.8 ^e^	>125.0 ^e^
Seed	EtOAc	15.4 ± 1.3 ^d^	7.5 ± 0.2 ^b^	86.1 ± 10.8 ^e,f^	138.7 ± 10.1 ^d^	>125.0 ^e^
	EtOH	265.1 ± 1.4 ^a^	6.8 ± 0.1 ^c^	2,228 ± 337 ^d^	492.8 ± 4.1 ^b^	16.0 ± 0.5 ^d^

NOTE: HEX = *n*-hexane extracts; EtOAc = ethyl acetate extracts; EtOH = ethanolic extract. The letters ^a–f^ denote significant difference among different *C. carandas* extracts (*p* < 0.05) after being analyzed by one-way analysis of variance (ANOVA) followed by Tukey’s test. ND refers to not determined.

**Table 2:** IC_20_ of each *C. carandas* extracts on U937 cells and RAW 264.7 cells.

*C. carandas* Extracts		IC_20_ (µg/mL)	
		U937 Cells	RAW 264.7 Cells
Ursolic acid		9.6 ± 1.1	8.2 ± 0.3
	Hex	>250	>100
Fruits	EtOAc	31.9 ± 6.7	15.9 ± 3.7
	EtOH	40.8 ± 3.5	>100
	Hex	>250	>100
Pulp	EtOAc	36.1 ± 8.0	20.9 ± 0.5
	EtOH	118.0 ± 12.6	>100
	Hex	>250	>100
Leaf	EtOAc	52.2 ± 3.7	15.0 ± 0.5
	EtOH	49.6 ± 3.5	>100
	Hex	>250	>100
Seed	EtOAc	7.4 ± 2.0	>100
	EtOH	34.6 ± 8.8	>100

**Note:** HEX = *n*-hexane extracts; EtOAc = ethyl acetate extracts; EtOH = ethanolic extract.

## Data Availability

Date is contained within the article.
